# Ovarian goiter detected during post-operative follow-up of papillary thyroid cancer: a case report

**DOI:** 10.1093/jscr/rjad012

**Published:** 2023-01-24

**Authors:** Daisuke Murayama, Soji Toda, Yoichiro Okubo, Hiroyuki Hayashi, Hiroyuki Iwasaki

**Affiliations:** Department of Breast and Thyroid Surgery, Aizawa Hospital, 2-5-1 Honjo, Matsumoto, Nagano 390-8510, Japan; Department of Breast and Endocrine Surgery, Kanagawa Cancer Center, 2-3-2 Nakao Asahi-ku, Yokohama, Kanagawa 241-8515, Japan; Department of Pathology, Kanagawa Cancer Center, 2-3-2 Nakao Asahi-ku, Yokohama, Kanagawa 241-8515, Japan; Department of Pathology, Yokohama Municipal Citizen’s Hospital, 1-1 Mitsuzawanishimachi Kanagawa-ku, Yokohama, Kanagawa 221-0855, Japan; Department of Breast and Endocrine Surgery, Kanagawa Cancer Center, 2-3-2 Nakao Asahi-ku, Yokohama, Kanagawa 241-8515, Japan

## Abstract

A 70-year-old female without any past medical history underwent total thyroidectomy and central neck dissection for papillary thyroid cancer (PTC) (pT3bN1aM0 pStage II). Her post-operative thyroglobulin (Tg) level remained high (around 100 ng/mL), which increased to 366 ng/mL 5 years after surgery. Computed tomography revealed metastasis to the left III and right Vb and VI lymph nodes and an incidental ovarian tumor. Transvaginal ultrasonography and magnetic resonance imaging suspected malignancy, resulting in total hysterectomy and bilateral adnexal resection. A pathological diagnosis of ovarian goiter with no malignancy was then established. For lymph node metastasis of PTC, right neck dissection and left III lymph node resection were performed. Post-operative blood examination showed a significant decrease in the Tg level (5.9 ng/mL). In conclusion, systemic imaging or I-131 remnant ablation should be performed after total thyroidectomy, as evident in the present case in which Tg levels did not decrease after total thyroidectomy.

## INTRODUCTION

Ovarian goiter (OG) is defined as an ovarian tumor with thyroid tissue occupying over 50% of the lesion, although, approximately, 15% of all teratomas contain a small, non-significant focus of thyroid tissue [1]. OG is a rare ovarian tumor that constitutes approximately 5% of all ovarian teratomas [2–4]. We herein report a case of post-operative lymph node recurrence of papillary thyroid cancer (PTC) coexistent with OG.

## CASE PRESENTATION

**Figure 1:**
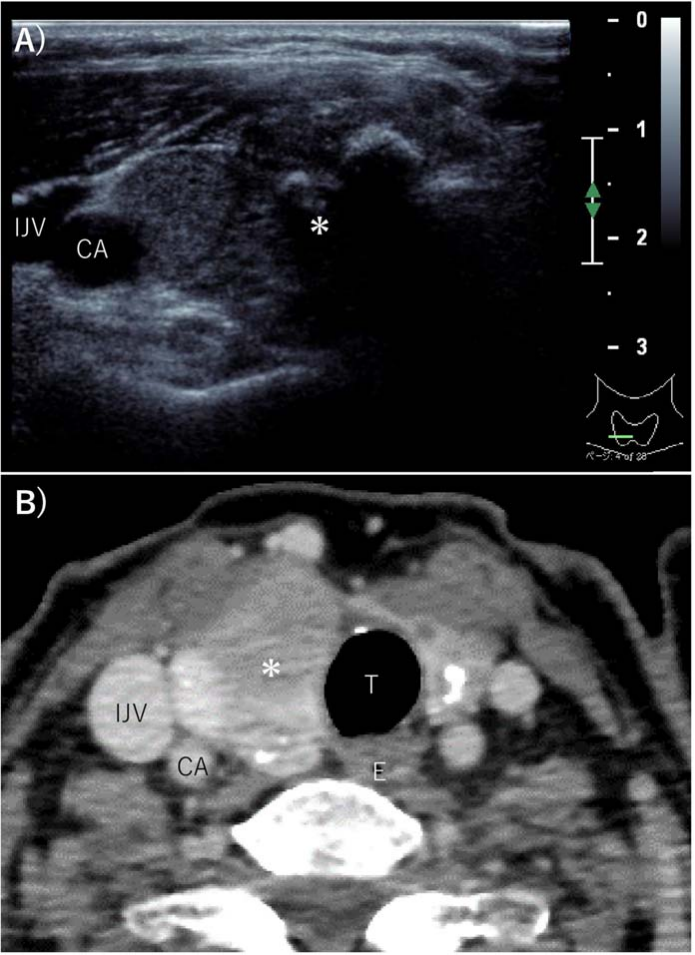
Pre-operative imaging findings of papillary thyroid cancer. (**A**) Ultrasonography revealed a hypoechoic 28-mm mass with internal calcification was found in the right lobe of the thyroid gland. (**B**) Contrast-enhanced CT revealed a 37 mm× 25 mm tumor in the right lobe of the thyroid gland without surrounding invasion and no lymph node metastasis or distant metastasis. ^*^: tumor, CA: carotid artery, IJV: internal jugular vein, T: trachea, E: esophagus.

**Figure 2:**
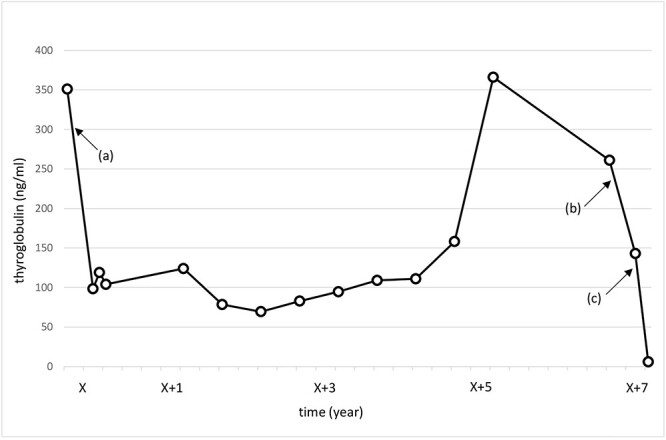
Change in the thyroglobulin level. X: date of initial surgery. (**a**) Total thyroidectomy and central neck dissection. (**b**) Total hysterectomy and bilateral adnexal resection. (**c**) Right neck dissection and left III lymph node resection.

**Figure 3:**
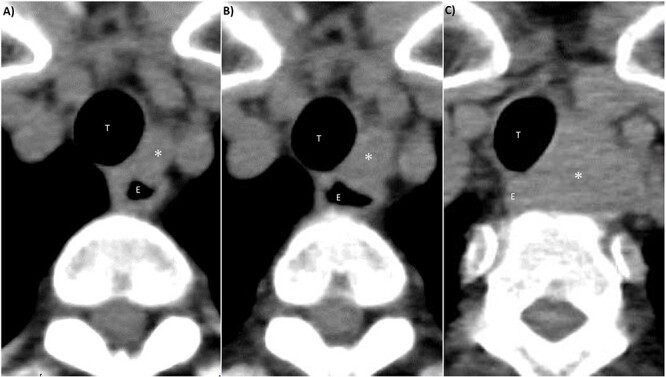
CT findings of left III lymph node metastasis. (**A**) 4 years after initial surgery, size: 9 mm × 7 mm. (**B**) 5 years after initial surgery, size: 14 mm × 10 mm. (**C**) 7 years after initial surgery, size: 32 mm × 27 mm. ^*^: left III lymph node, T: trachea, E: esophagus.

**Figure 4:**
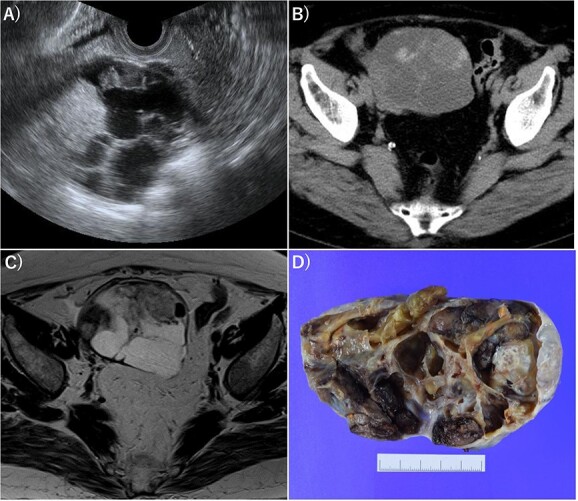
Imaging of OG. (**A**) Transvaginal ultrasonography showed numerous separate structures and substantial nodules. (**B**) CT showed a 10.5-cm multifocal cystic tumor on the right side of the uterus. (**C**) Magnetic resonance imaging (T2-weighted image) revealed a tumor with numerous separate structures and substantial nodules, which led to speculations that the cystic area was serous. Right ovarian cancer was suspected. (**D**) Gross examination also showed numerous separate structures and substantial nodules.

**Figure 5:**
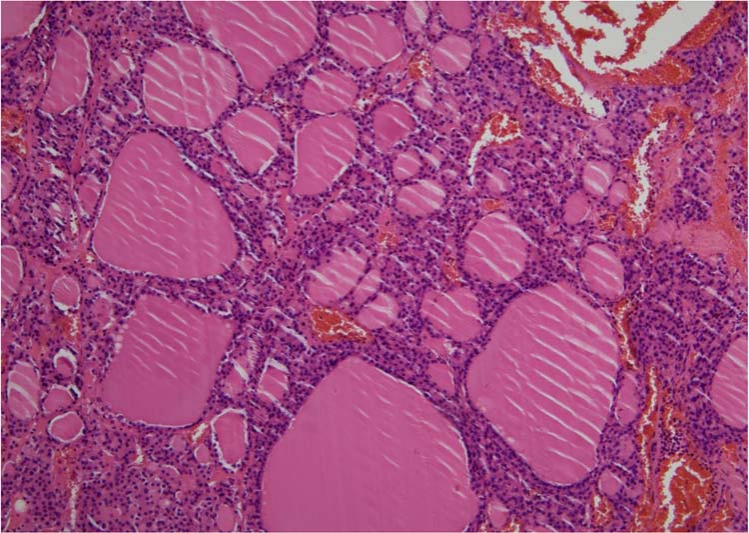
Pathological diagnosis of OG was established (HE, ×100). Histologically, the right ovarian tumor showed large and small thyroid follicles with colloid, no papillary thyroid carcinoma nuclei and no malignant findings.

A 70-year-old female without any past medical history underwent total thyroidectomy and central neck dissection for PTC (pT3bN1aM0 pStage II; Fig. 1). Pre-operative blood examination showed a thyroglobulin (Tg) level of 351 ng/mL (normal value of Tg, ≤33.7 ng/mL). After surgery, her Tg level remained around 100 ng/mL (Fig. 2). However, 5 years after initial surgery, her Tg level increased to 366 ng/mL, with subsequent computed tomography (CT) revealing metastasis to the left III (Fig. 3) and right Vb and VI lymph nodes. CT also incidentally observed a right ovarian tumor, for which transvaginal ultrasonography and magnetic resonance imaging (MRI) were performed given suspicions of malignancy (Fig. 4). Blood examination showed a CA125 level of 33.4 U/mL (normal value of CA125, ≤35.0 U/ml). Given suspicions of malignancy by our hospital’s department of gynecology, total hysterectomy and bilateral adnexal resection had been conducted. A pathological diagnosis of OG with no malignancy was then established (Fig. 5). The patient’s post-operative Tg level decreased to 143 ng/mL (Fig. 2). For lymph node metastasis of PTC, right neck dissection and left III lymph node resection by sternal incision were performed. The left recurrent laryngeal nerve was resected due to the invasion of the left III lymph node. Though post-operative swallowing rehabilitation was needed, she was discharged 9 days after surgery with good clinical course. Post-operative blood examination showed a significant decrease in the Tg level (5.9 ng/mL).

## DISCUSSION

OG is defined as an ovarian tumor with thyroid tissue occupying over 50% of the lesion, although, approximately, 15% of all teratomas contain a small, nonsignificant focus of thyroid tissue [1]. OG is a rare ovarian tumor that constitutes approximately 5% of all ovarian teratomas [2–4]. The median age at diagnosis of OG was 46 years [5]. OG had been firstly described in 1889 by Boettlin who observed the presence of thyroid follicular tissue in ovaries [6]. Malignancy accounts for approximately 5% of OG, with the most common malignancy being PTC [5, 7]. Moreover, metastases have been detected in 5–23% of patients with malignant OG [8]. Wei *et al*. reported that nine cases presented with no recurrence except for one with infiltration of the round ligament and speculated that resection of malignant OG offers good prognosis [5]. Evidence has shown that the most common symptoms of OG included a palpable lower abdominal mass (23.5%), followed by lower abdominal pain (20.6%) and abnormal vaginal bleeding (8.8%), with 41.2% of patients presenting no specific symptoms [4]. Rarely, 5–8% of patients with OG are diagnosed hyperthyroidism [3, 9–12]. Moreover, images obtained via ultrasonography, CT and MRI can often be similar to those observed in ovarian cancer, especially in cases with thickening of a peripheral cyst wall or septum [13]. Ascites has been reported in 15–20% of patients with OG [14], with a few cases also presenting plural effusion, resulting in pseudo-Meigs syndrome [15]. Although serum CA125 levels are often elevated in ovarian cancer, it can also be increased in OG [15]. In fact, Paladini *et al*. reported a case of OG with a serum CA125 level of 2548 U/mL [15]. Diagnosing OG pre-operatively has been considered challenging. Notably, Fujie *et al*. reported that I-131 had a sensitivity and specificity of 61 and 98%, respectively, for diagnosing ectopic and malignant thyroid tissues [16]. Had I-131 remnant ablation been performed after the initial surgery for PTC in our case, OG might be revealed. As such, there is a need to consider the possibility of ectopic thyroid tissues, such as OG or distant metastasis.

In conclusion, systemic imaging or I-131 remnant ablation should be performed after total thyroidectomy, as evident in the present case in which Tg levels did not decrease after total thyroidectomy.

## Data Availability

The datasets used during the current study are available from the corresponding author upon request.
